# An annotated wing interferential pattern dataset of dipteran insects of medical interest for deep learning

**DOI:** 10.1038/s41597-023-02848-y

**Published:** 2024-01-02

**Authors:** Arnaud Cannet, Camille Simon-chane, Aymeric Histace, Mohammad Akhoundi, Olivier Romain, Marc Souchaud, Pierre Jacob, Darian Sereno, Philippe Bousses, Denis Sereno

**Affiliations:** 1 Direction des affaires sanitaires et sociales de la Nouvelle-Calédonie, Nouméa, France; 2 ETIS UMR 8051, Cergy Paris University, ENSEA, CNRS, F-95000 Cergy, France; 3 Parasitology-Mycology, Hopital Avicenne, AP-HP, Bobigny, France; 4 Univ. Bordeaux, CNRS, Bordeaux INP, LaBRI, UMR 5800, F-33400 Talence, France; 5 InterTryp, Univ Montpellier, IRD-CIRAD, Infectiology, Entomology and One Health Research Group, Montpellier, France; 6 MIVEGEC, Univ Montpellier, CNRS, IRD, Montpellier, France

**Keywords:** Biodiversity, Entomology

## Abstract

Several Diptera species are known to transmit pathogens of medical and veterinary interest. However, identifying these species using conventional methods can be time-consuming, labor-intensive, or expensive. A computer vision-based system that uses Wing interferential patterns (WIPs) to identify these insects could solve this problem. This study introduces a dataset for training and evaluating a recognition system for dipteran insects of medical and veterinary importance using WIPs. The dataset includes pictures of Culicidae, Calliphoridae, Muscidae, Tabanidae, Ceratopogonidae, and Psychodidae. The dataset is complemented by previously published datasets of Glossinidae and some Culicidae members. The new dataset contains 2,399 pictures of 18 genera, with each genus documented by a variable number of species and annotated as a class. The dataset covers species variation, with some genera having up to 300 samples.

## Background & Summary

Blood-sucking insects, such as mosquitoes, ticks, and sandflies, transmit viral, parasitic, or bacterial pathogens that cause severe diseases, including arboviruses, malaria, Lyme disease, and others. Climate fluctuations, global economic growth, migration, and increased trade are factors that influence the distribution of many organisms, not just insects. The expansion of the tiger mosquito, *Aedes albopictus,* into new climates, which it has recently done, is a concern as it is an established vector for Zika, chikungunya, and dengue viruses^1^. To address the threats of emerging vector-borne diseases, robust and rapid species identification is crucial. However, current global vector surveillance systems are unstandardized and facing a global shortage of entomologists. Identification is typically conducted by skilled personnel using quantitative and qualitative criteria, such as specimen size, shape, texture, or the presence or absence of certain key features. Nevertheless, when sympatric species have medical importance, these distinctions based on morphological characteristics may not always be discriminative. Additionally, older adult specimens may have missing or damaged body parts or characters essential for exact identification. These samples can also have damage in critical diagnostic character regions, making it challenging to separate vector species from closely related non-vector ones. Finally, the identification of dipteran species is complex and requires highly specialized expertise if diversity is to be fully addressed.

The identification of specimens at the species/subspecies level is crucial during proactive surveys to address health risks associated with their introduction or presence. However, morphological criteria are inadequate when samples are damaged or for extensive geographic surveys, and identification methods based on heavy biological protocols (DNA and mass spectrometry) are expensive, incompatible with in-field analyses, and destructive to samples, including pathogens. Therefore, fine-grain, non-destructive entomological surveillance methods that allow for later pathogen identification with high efficiency, accuracy, and reduced costs are needed. Guidelines for mosquito surveillance are publicly available^2^.

Thin-film interference generated on the transparent wings with a thin membrane allows the formation of a colored pattern. With incoming external light wings in light-absorbing and dark environments, WIPs are displayed on the wing membranes. These WIPs vary significantly among species but faintly between specimens of the same species or between sexes. Since the 2010s, WIPs (Wing Interference Patterns) have received significant attention for their potential as a method for species identification^3–5^. The role of WIPs in sexual selection of Drosophila melanogaster is such that males with more vivid wings are more attractive to females than to males with dull wings^6^. This enhances the visual aspect of the mating tool array of Drosophila. Unlike iridescence, which depends on the angle of a flat film, wing structures act as diopters, making WIPs appear non-iridescent^4^. The Newton color series observed on wings resembles that of a soap bubble and is proportional to the wing membrane thickness at any given point, which helps in species identification^7,8^. Collecting colored patterns is relatively easy, and deep learning-trained descriptors extracted from pictures have demonstrated exceptional accuracy in identifying insect species^9–13^. The image dataset, raw or processed, combined with already publicaly available ones on Glossinidae, Some Culicidae members can serve as an authenticated dataset for recognizing seven families or twenty-one genera, including those with medical or veterinary interests, and can be utilized by users such as machine learning engineers, app developers, data scientists, taxonomists, and medical and veterinary entomologists.

## Methods

This method has been previously used, on Glossinidae and some Culicidae members, and results of the identification process were published^11–13^. Here, we provide dataset that complete other previously published to expand it and on which the procedure can be applied to dipteran insects belonging to 7 families (Culicidae, Calliphoridae, Muscidae, Glossinidae, Tabanidae, Ceratopogonidae, Psychodidae) and 21 genera. The method consists of selecting insects from 7 families; whenever possible, at least ten specimens, including male and female ones, were chosen for being included in the database. Then, wings are dissected, and WIPs are captured to fill the database. The automatic classification was performed as previously described^9,11–13^ using a larger dataset of Dipteran insects collected during this study^14^.

### Resources of specimen

The insect specimens were gained from ARIM collection belonging to IRD (Institut de Recherche pour le Développement) (https://arim.ird.fr/) from well-established laboratory-reared or field-caught specimens. The ARIM collection kept more than 100,000 preserved and stored insect specimens.

### Data collection

**Fig. 1:**
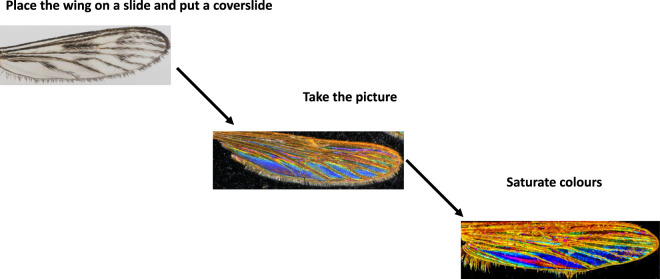
Schematic representation of the image acquisition and processing, example of *Culiseta* samples.

Insect wings were dissected and deposited on a glass slide. Samples preserved in 70° ethanol were layered overnight at room temperature on a glass slide before being processed. A cover slide is deposited on the sample before image acquisition. The picture are taken with a Keyence™ VHX 1000 microscope, with the VH-Z20r camera and a VH K20 adapter, an illumination incidence of 10°. Image acquisition was performed using the High Dynamic Range (HDR) function. Magnification was adjusted to ensure constant-size pictures; a schematic representation of the process and output is given in Fig. 1.

The numerical parameter settled were as follow:

- Camera White Balance: 3200 K
- Shutter Speed: preset 1/15(sec)
- Gain: 0 dB
- Frame rate 15 F/s

HDR function:

- Brightness: 15%
- Texture: 15%
- Contrast: 45%
- Color: 100%

Next, the luminosity, contrast, shadow, reflection, and saturation were settled at 80, 100, 0, 0, and 100%, respectively, using Window 7 familial edition. Table 1 show examples of processed image for dataset implementation.

**Table 1:** Illustration of the current dataset diversity regarding families, genera, and specimens.

Label		
Family	Genera	
Callyphoridae	*Auchmeromyia*	
	*Chrysomyia*	
	*Hemipyrelia*	
	*Lucilia*	
	*Tryciclea*	
Ceratopogonidae	*Culicoides*	
Culicidae	*Coquillettidia*	
	*Culex*	
	*Culiseta*	
	*Lutzia*	
	*Orhtopodomyia*	
Muscidae	*Haematobia*	
Psychodidae	*Lutzomyia*	
	*Phlebotomus*	
	*Sergentomyia*	
Tabanidae	*Atylotus*	
	*Chrysops*	
	*Tabanus*	

## Data Records

**Fig. 2:**
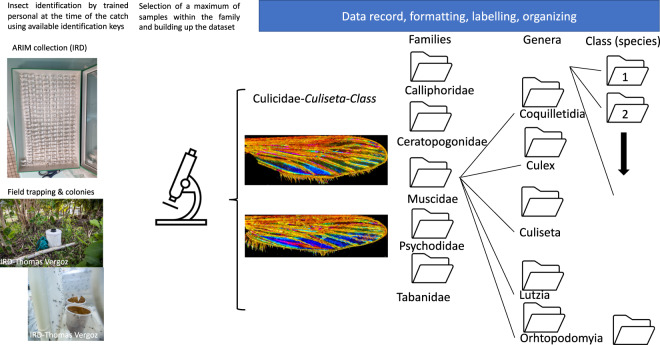
Schematic representation of the image acquisition and labeling workflow.

The dataset is publicly available in Figshare^14^. Figure 2 illustrates the workflow to record and organize it. Specimens belonging to the Culicidae (*Culiseta annulata*) family were used as examples to demonstrate the process, all samples being processed according to the same workflow. Only specimens displaying wing integrity >60% (arbitrarily set) with a distinguishable Wing Interferential Pattern are filled in the database. The sole exception is the Tabanus specimen, which doesn’t display a distinguishable WIP.

**Fig. 3:**
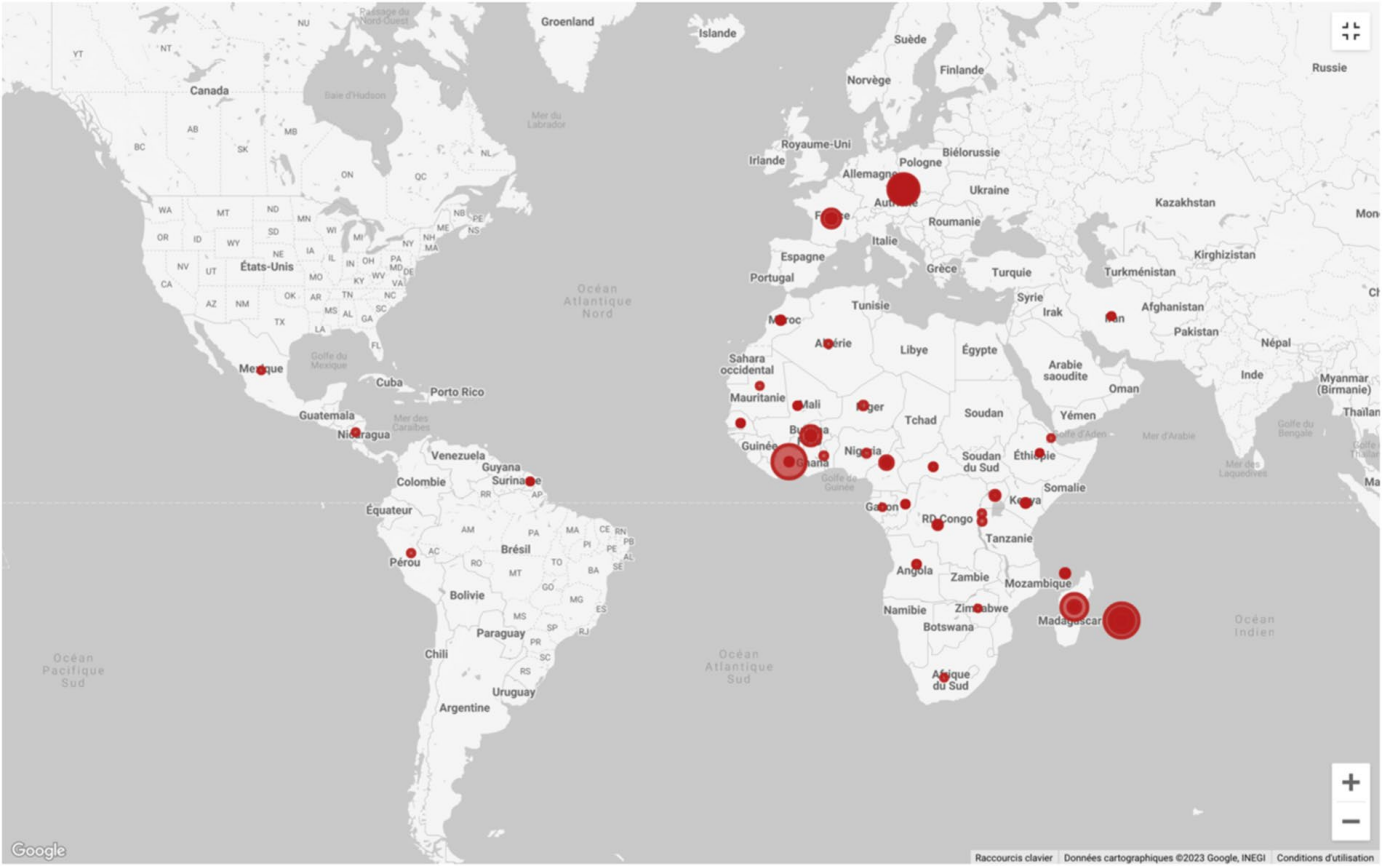
Geographic distribution of samples using Google Looker Studio (https://lookerstudio.google.com/overview).

The origin of samples is presented in Fig. 3; note that the geographic origin of specimens from laboratory-reared colonies is not representative of the original one.

The geographic distribution depicts that most samples originate from Africa, Madagascar, and La Reunion Island. Most specimens having a European origin are colony-reared ones.

For further species-level identification (species recognition system), the images were organized in individual specimens in the genus folder of the dataset. Spreadsheets are organized as follows: numeration of the picture, Order, family, Genus, and Class. Each class corresponds to an individual species see Fig. 2. In the dataset, the unique image of Tabanus wing filled doesn’t display WIPs, and efforts must be engaged to gather them.

## Technical Validation

### Taxonomy

The identification of insects at the genus, species, or subspecies level was performed by trained entomologists using the available keys at the time of their catch. Only the adult stage was used for WIPS image acquisition.

### A pilot test

The method has been validated in our previous work^9,11–13^

## Usage Note

### Usage of the dataset

1. Entomologists can use the dataset gathering 2,399 pictures of 18 genera, for training for taxonomic and/or machine learning engineers.
2. Combining the dataset repository provided in this study^14^ with the previously published dataset^15^ allow to extend the diversity to 5516 pictures of 7 families (Culicidae, Calliphoridae, Muscidae, Glossinidae, Tabanidae, Ceratopogonidae Psychodidae) and 21 genera. See the Table 2 for Family, Genus and picture number filled in each dataset.

**Table 2:** Families and genera of WIPs pictures included in the datasets.

Label (Number of pictures)		
Family	Genera	Repository reference
Calliphoridae (35)	*Auchmeromyia* (3)	^14^
	*Chrysomyia* (7)	^14^
	*Hemipyrelia* (5)	^14^
	*Lucilia* (6)	^14^
	*Tryciclea* (14)	^14^
Ceratopogonidae (28)	*Culicoides* (28)	^14^
Culicidae (1992)	*Aedes* (502)	^15^
	*Anopheles* (849)	^15^
	*Coquillettidia* (5)	^14^
	*Culex* (591)	^14^
	*Culiseta* (13)	^14^
	*Lutzia* (14)	^14^
	*Orhtopodomyia* (18)	^14^
Glossinidae (1766)	*Glossina* (1766)	^15^
Muscidae (4)	*Haematobia* (4)	^14^
Psychodidae (1673)	*Lutzomyia* (294)	^14^
	*Phlebotomus* (1272)	^14^
	*Sergentomyia* (107)	^14^
Tabanidae (18)	Atylotus (5)	^14^
	Chrysops (12)	^14^
	Tabanus (1)	^14^

Limitations of the dataset:

1. The dataset consists of imbalanced classes (species) of images due to difficulties in gathering enough specimens because we cannot gather them in the ARIM database, we do not get financial resources to collect them in natura, or there are no colonies available.
2. The dataset does not represent the whole family/Genera/species diversity of dipteran insects of medical and veterinary interest.
3. Be aware that images in the dataset were resized, computed, and processed in terms of luminosity, contrast, shadow, reflection, and saturation, which is limiting for applications requiring wing thickness measurement deduced from Newton color series
4. The eligibility criteria for data inclusion in the dataset are not restrictive; damaged samples were included that might be limiting for some application

## Data Availability

The source code is publicly available in GitHub, with a direct URL: https://github.com/marcensea/diptera-wips.git.
